# Cardiovascular characteristics of patients initially diagnosed breast cancer

**DOI:** 10.1186/s13019-021-01608-6

**Published:** 2021-08-12

**Authors:** Zhaoying Dong, Fan Zhang, Qiaojuan Huang, Zhaojun Liu, Siyu Chen, Tao Xu, Jun Xiao, Changhong Zhang, Xiaoli Zhou

**Affiliations:** 1 Department of Cardiology, The First Affiliated Hospital of Chongqing Medical University, 1 Youyi Road, Yuzhong District, Chongqing, 400000 China; 2 School of Public Health and Management, Research Center for Medicine and Social Development, Collaborative Innovation of Chongqing Medical University, Chongqing, 400000 China; 3 Department of Endocrine and Breast Surgery, The First Affiliated Hospital of Chongqing Medical University, Chongqing, 400000 China

**Keywords:** Breast cancer, Cardiovascular risk factors, Axillary lymph node metastasis, Initial diagnosis

## Abstract

**Objective:**

We aimed to explore the cardiovascular characteristics of patients who were initially diagnosed with breast cancer.

**Methods:**

A total of 600 patients who were diagnosed with primary breast cancer were included in this retrospective study. The data of fasting blood glucose, total cholesterol, total triglyceride, high-density lipoprotein cholesterol, low-density lipoprotein cholesterol, lipoprotein (a) (LP (a)) and serum uric acid were collected. Univariate analysis was used to evaluate the cardiovascular risk factors (CVRFs) in patients with breast cancer. The arteriosclerotic cardiovascular disease (ASCVD) risk assessment was performed. Multivariate analysis was used to identify the factors that influenced axillary lymph node metastasis (ALNM).

**Results:**

Compared with the premenopausal group, the prevalence of overweight/obesity (47.6% vs. 35.2%), diabetes (12.8% vs. 4.3%) and hypertension (49.7% vs. 26.3%) were significantly increased in the postmenopausal group (*p* < 0.05). Comparisons of rural patients and urban patients showed that there were significant differences in the diagnostic age (49.94 ± 9.92 vs. 52.59 ± 11.13) in the rural patients was notably younger in comparison with the urban patients (*p* < 0.05). However, the number of menopausal patients (44.3% vs. 53.3%) in the rural group were decreased in comparison with the urban group (*p* < 0.05). In ASCVD risk stratification, the proportion of low-risk patients (56.4% vs. 90.8%), medium-risk patients (20.6% vs 0.3%) and high-risk patients (19.3% vs. 6.6%) were significantly different between the postmenopausal group and premenopausal group (*p* < 0.05). Residence (OR 0.735; 95% CI 0.516–1.046; *p* = 0.087), the number of children (OR 1.250; 95% CI 0.990–1.578; *p* = 0.061) and LP (a) of ≥ 500 mg/L (OR 0.603; 95% CI 0.342–1.063; *p* = 0.080) were independent influencing factors of ALNM.

**Conclusion:**

Postmenopausal patients have more CVRFs and higher risks of ASCVD than premenopausal patients initially diagnosed with breast cancer. There was a correlation between CVRFs and ALNM in patients with breast cancer.

## Introduction

Breast cancer is the most common malignant tumor among women worldwide. It is currently estimated that there are approximately 1.6 million new breast cancer cases worldwide in 2012 [1]. However, the incidence and mortality rates vary substantially, and the 5-year survival rate can reach 85% to 90% in the well-developed regions of the world [2]. It has been reported that patients with breast cancer have a significantly increased risk of cardiovascular disease (CVD) in comparison with the healthy controls [3]. The mortality rate of elderly breast cancer patients due to the complications of CVD and cerebrovascular disease is as high as 15.9%, which is higher than that due to the recurrence of breast cancer (15.1%) [4]. CVD has become the main cause of death in breast cancer patients [5]. Cancer and CVD have entered into a ‘clinical overlap’ era [6].

The patients with breast cancer who also have cardiovascular risk factors (CVRFs) are more likely to develop drug-related cardiovascular problems after chemotherapy with anthracycline [7]. Additionally, radiotherapy and chemotherapy significantly increased the incidence of CVD and mortality of patients with breast cancer [8]. Therefore, it is necessary to understand the cardiovascular status before treatment. At present, there are many problems in most studies on CVRFs of patients with breast cancer at home and abroad, such as a lack of clinical data and incomplete data acquisition. Therefore, it is difficult to comprehensively analyze CVRFs of patients with breast cancer. The purpose of this study was to investigate the CVRFs and arteriosclerotic cardiovascular disease (ASCVD) risk assessment in patients initially diagnosed breast cancer in Southwest China.

## Subjects and methods

### Subjects

A total of 600 patients diagnosed with breast cancer at the Breast Cancer Center in the First Affiliated Hospital of Chongqing Medical University from January 2014 to December 2019 were included in this retrospective study. Depending on whether menopause had occurred when breast cancer was diagnosed, patients were divided into the premenopausal group (n = 304) and postmenopausal group (n = 296). According to whether there was axillary lymph node metastasis (ALNM), patients were divided into metastasis group and a non-metastasis group. Inclusion criteria: (1) female patients with primary breast cancer; (2) patients with clear histological type in pathological report; (3) patients with original medical record in which there were the descriptions of primary tumor, axillary lymph node and distant metastasis. (4) Patients did not receive neo-adjuvant chemotherapy. Exclusion criteria: (1) patients with type I diabetes, nonessential hypertension; (2) patients with a history of malignant cancer at diagnosis or a history of hysterectomy; (3) male patients; (6) patients with distant metastasis at diagnosis; (4) patients with a history of severe hepatic and renal dysfunction; and (5) patients with abnormal thyroid function.

### Data collection

Routine examination data of patients diagnosed with primary breast cancer were collected, including general information, auxiliary inspection information, etc. Fasting blood glucose (FBG), total cholesterol (TC), total triglyceride (TG), high-density lipoprotein cholesterol (HDL-C), low-density lipoprotein cholesterol (LDL-C), lipoprotein (a) (LP (a)) and serum uric acid (SUA) were measured at the Medical Examination Center in the First Affiliated Hospital of Chongqing Medical University. Whether there was the presence of ALNM was collected from the pathological report in the pathology center.

### Diagnostic criteria

**Fig. 1:**
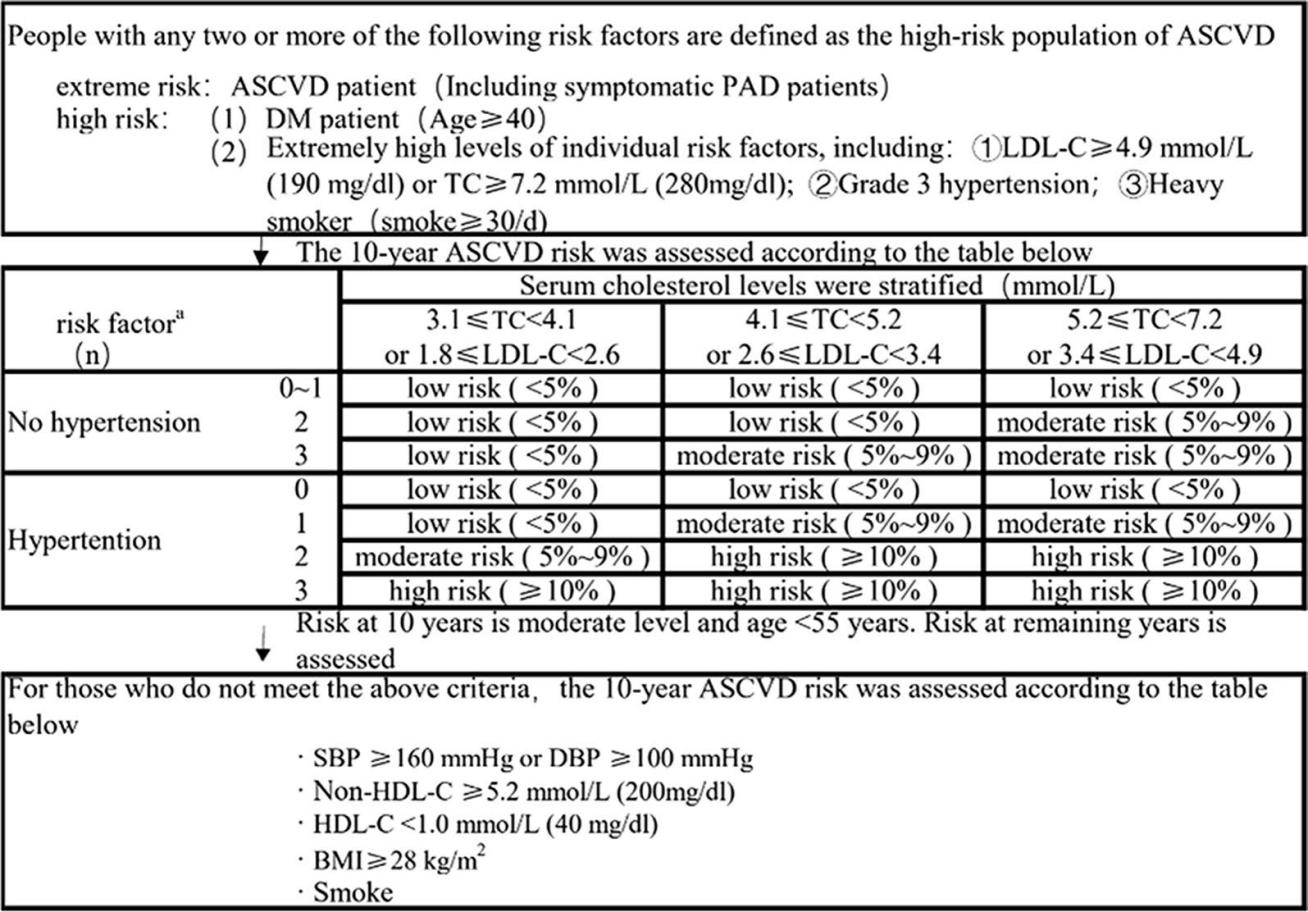
ASCVD risk stratification

Diabetes[9]: fasting glucose ≥ 7.0 mmol/L, randomized glucose ≥ 11.1 mmol/L, or oral glucose tolerance test (OGTT) 2-h glucose ≥ 11.1 mmol/L. Hypertension [10]: Blood pressure ≥ 140/90 mmHg in the consultation room (blood pressure in the consultation room was repeatedly measured for three times). Hyperlipidemia [11]: TC ≥ 6.2 mmol/L, TG ≥ 2.3 mmol/L, HDL-C < 1 mmol/L or LDL-C ≥ 4.1 mmol/L. Hyperuricemia [12]: Fasting blood uric acid levels were observed twice on different days on a normal purine diet, and it is of > 420 µmol/L (7.06 mg/dL) without gout attack for Chinese adults. Body mass index [13]: The limit for overweight is BMI = 24 and that for obesity is BMI ≥ 28 for Chinese adults. LP (a) [14]: LP (a) of ≥ 500 mg/L is used as a powerful ASCVD risk threshold. Menopause [15]: If 12 months after the last menstrual period, there is still no menstrual period, the patient could be clinically diagnosed with menopause after the exclusion of the possibility of pregnancy. The ASCVD risk stratification is shown in Fig. 1.

### Statistical analysis

SPSS 26.0 statistical software was used for statistical analysis. For univariate analysis, graded variables and categorical variables were compared using chi-squared tests, and *p* < 0.05 indicated a significant difference. The continuous variable Age, a continuous variable, was normally distributed, and an independent sample test was used for analysis. Variables were represented as means ± standard deviation (SD). The remaining data were analyzed with descriptive statistics. Age, overweight/obesity, diabetes, hypertension, hyperlipidemia, LP (a), hyperuricemia, ALNM, the place of residence, and the number of children, menstrual status were analyzed by univariate logistic regression analysis to identify the factors affecting ALNM. Then, binary logistic regression analysis was performed to identify factors influencing ALNM in patients with breast cancer. The backward elimination was used to identify variables that were independent risk factors, and P < 0.1 was considered to indicate a significant difference.

## Results

### The basic characteristics

**Fig. 2:**
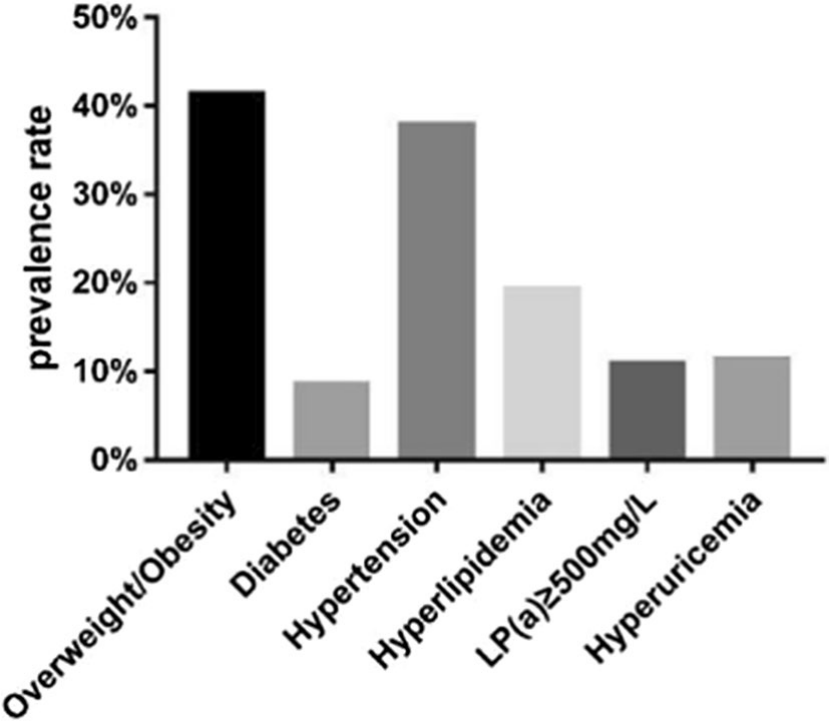
The overall combination of CVRFs

Among the 600 patients with primary breast cancer, their age was (51.43 ± 10.68) years (ranging from 26 to 90 years). A total of 304 patients (50.67%) were premenopausal, and 296 (49.33%) were postmenopausal. A total of 338 patients (56.33%) lived in the city, and 262 (43.67%) lived in the country. Furthermore, 562 patients (93.67%) had a history of childbearing, and 38 (6.33%) had no history of childbearing. 248 patients (41.33%) were overweight or obese, and 51 (8.5%) had diabetes mellitus. There were 227 patients (37.83%) with hypertension, 116 (19.33%) with a combination of hyperlipidemia, and 65 (10.83%) with LP (a) ≥ 500 mg/L. Additionally, 68 patients (11.33%) were found to have hyperuricemia (Fig. 2).

### Combination of CVRFs in premenopausal and postmenopausal patients with breast cancer in different living environments (city/country)

There were statistically significant increase in the prevalence of overweight/obesity, type 2 diabetes, and hypertension in the postmenopausal patients in comparison with the premenopausal patients (*p* < 0.05) (Table 1). Rural patients with breast cancer were first diagnosed at a younger age than those living in cities, and the number of rural postmenopausal patients with breast cancer was significantly lower than the number of urban patients (*p* < 0.05) (Table 2).

**Table 1:** Characteristics of patients with initial breast cancer [n(%)]

Characteristics	All (n = 600)	Premenopausal group (n = 304)	Postmenopausal group (n = 296)	T/χ^2^	*p*
Age	51.43 ± 10.68	43.72 ± 6.68	59.360 ± 7.868	-26.220	*0.000*
Overweight/obesity	248 (41.33)	107 (35.20)	141 (47.64)	9.568	*0.002*
Type 2 diabetes	51 (8.50)	13 (4.28)	38 (12.84)	14.134	*0.000*
Hypertension	227 (37.83)	80 (26.32)	147 (49.66)	34.755	*0.000*
Hyperlipidemia	116 (19.33)	50 (16.45)	66 (22.30)	3.291	0.070
LP (a)				0.594	0.441
≤ 500 mg/L	535 (89.17)	274 (90.13)	261 (88.18)		
> 500 mg/L	65 (10.83)	30 (9.86)	35 (11.82)		
Hyperuricemia	68 (11.33)	27 (8.88)	41 (13.85)	3.686	0.055
Axillary lymph node metastasis	262 (48.25)	129 (46.07)	133 (50.57)	1.099	0.294

*BMI* Body Mass Index, *LP (a)* Lipoprotein (a)

**Table 2:** Characteristics of urban and rural patients with initial breast cancer [n(%)]

Characteristics	Urban group (n = 338)	Rural group (n = 262)	T/χ^2^	*p*
Age	52.59 ± 11.13	49.94 ± 9.92	3.027	*0.003*
Postmenopausal	180 (53.25)	116 (44.27)	4.761	*0.029*
Overweight/obesity	131 (38.76)	117 (44.66)	2.118	0.146
Type 2 diabetes	33 (9.76)	18(6.87)	1.588	0.208
Hypertension	137 (40.53)	90(34.35)	2.398	0.122
Hyperlipidemia	63 (18.64)	53 (20.23)	0.239	0.625
LP (a)			0.183	0.669
≤ 500 mg/L	303 (89.64)	232 (88.55)		
> 500 mg/L	35 (10.36)	30 (11.45)		
Hyperuricemia	9 (2.66)	6 (2.29)	0.084	0.772
Axillary lymph node metastasis	140 (41.42)	122 (46.56)	3.050	0.081
Missing	27 (Of the total)	30 (Of the total)		

*BMI* Body Mass Index, *LP (a)* Lipoprotein (a)

### ASCVD risk stratification of patients with breast cancer

**Fig. 3:**
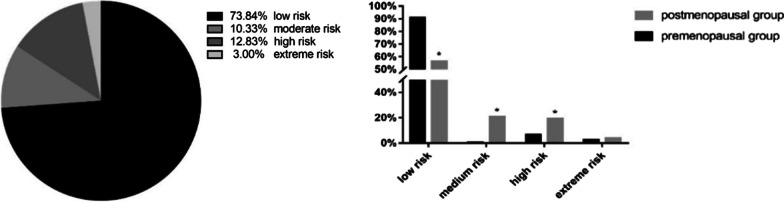
ASCVD risk stratification of patients with breast cancer. **a** ASCVD risk stratification analysis. **b** ASCVD risk stratification analysis in premenopausal and postmenopausal breast cancer patients

A total of 18 patients (3%) had an extreme risk of ASCVD, 77 (12.83%) had a high risk of ASCVD, 62 (10.33%) had a moderate risk of ASCVD in the next 10 years, and 443 (73.84%) had a low risk (Fig. 3a). ASCVD risk stratification analysis in the premenopausal and postmenopausal patients with breast cancer showed that in the low-risk group, the number of premenopausal patients was significantly higher than the number of postmenopausal patients (*p* < 0.05). There were significant increase in the number of postmenopausal patients in the moderate-risk and high-risk groups than the number of premenopausal patients (*p* < 0.05) (Fig. 3b).

### Correlation analysis of CVRFs and ALNM in patients with breast cancer

Univariate and multivariate analyses of ALNM in patients with breast cancer were shown in Table 3. Univariate logistic analysis showed that in addition to the place of residence, the number of children and LP (a) of ≥ 500 mg/L were significantly associated with ALNM (*p* < 0.1). Binary logistic regression analysis showed that the independent influencing factors for ALNM were the place of residence (*p* = 0.087), number of children (*p* = 0.061) and LP (a) ≥ 500 mg/L (*p* = 0.080). Further analysis showed that patients with breast cancer combined with LP (a) level of ≥ 500 mg/L were more likely to have ALNM than breast cancer patients with LP (a) levels < 500 mg/L op to 0.63 times. Patients living in rural areas were more likely to have ALNM than those living in cities up to 0.74 times. For each additional child, the risk of ALNM was 1.25 times higher.

**Table 3:** Multivariate analysis of factors related to axillary lymph node metastasis in patients with breast cancer

Items	Univariate		*p*-values	Multivariate		*p*-values
	OR	95% CI		OR	95% CI	
Age	1.012	0.996–1.028	0.145	1.006	0.981–1.032	0.632
Place of residence	0.738	0.525–1.038	0.081	0.735	0.516–1.046	0.087
Number of children	1.279	1.014–1.613	0.038	1.250	0.990–1.578	0.061
Menstrual status	0.835	0.596–1.170	0.295	0.951	0.568–1.590	0.847
Overweight/obesity	1.082	0.769–1.524	0.650			
Diabetes	1.065	0.585–1.940	0.836			
Hypertension	0.905	0.641–1.278	0.570			
Hyperlipidemia	1.155	0.757–1.763	0.504			
LP (a) ≥ 500 mg/L	0.578	0.332–1.008	0.053	0.603	0.342–1.063	0.080
Hyperuricemia	0.575	0.186–1.781	0.338			

## Discussion

With the increasing incidence of breast cancer and the increasing long-term survival, CVD has become the leading cause of death in patients with breast cancer. Breast cancer and CVD have many common risk factors, and they affect each other. Additionally, chemotherapy, radiotherapy and endocrine therapy further affect the cardiovascular health of patients with breast cancer. Therefore, it is necessary to know the combination of CVRFs in patients with breast cancer diagnosed for the first time. The early management and intervention of CVD in breast cancer patients is beneficial.

CVD is increasingly recognized as an important factor affecting the survival rate and quality of life of patients with breast cancer [16, 17]. Patients with breast cancer have significantly higher risks of CVD than the healthy controls [18]. Among the 600 patients in our study, the proportion of adult female patients with T2DM is lower than that of female patients without T2DM [23]. This may be related to the exclusion of patients who had received medication for diabetes before the diagnosis of breast cancer. Additionally, oral glucose tolerance test (OGTT) screening has not been performed on patients with normal fasting blood glucose (FBG) levels, and occult diabetes and prediabetes are often missed. However, the proportion of postmenopausal patients with breast cancer and T2DM is still higher than that of patients with DM in China, which is worthy of attention [19]. The prevalence of DM is increased with age. Patients with early T2DM will develop hyperinsulinemia due to insulin resistance. The insulin/insulin receptor (IR) signaling pathway plays an important role in breast cancer progression by stimulating the RAS/RAF/MAPK kinase/ERK cascade in breast tissues, which results in tumor cell proliferation, survival and migration [20, 21]. In our study, the prevalence of overweight/obesity in postmenopausal patients with breast cancer is significantly higher than that in the premenopausal patients. This is consistent with the findings of Marian L Neuhouser et al., they find that the presence of overweight or obesity increases the risk of breast cancer in postmenopausal women [19]. Fat and inflammation promote the progression of breast cancer, and persistently high estrogen levels increase the risk of breast cancer. Overweight/obese and T2DM individuals have a large amount of fat accumulation, and adipose tissue can produce inflammatory factors and synthesize endogenous estrogen [22]. Therefore, special attention should be paid to active breast cancer screening in postmenopausal women with overweight/obesity and T2DM.

In the present study, the prevalence of hypertension in patients with breast cancer is 27.2%, which is higher than that in Chinese adult women [23]. Breast cancer and hypertension share a common pathophysiological pathway, which is due to that inflammation that induces the occurrence of cancer may also induce hypertension [24]. It may be linked to the blocking and altering of apoptosis by hypertension [25]. However, pathways involved in the relationship between hypertension and breast cancer are still unclear [26]. In our study, the prevalence of hypertension in postmenopausal patients with breast cancer is higher than that in premenopausal women, and the prevalence in China is also higher than other Asian countries (30.60%)[27]. Our results are consistent with the increasing prevalence of hypertension with increasing age, and it may also be related to high salt intake in Southwest China.

The 10-year ASCVD risk assessment form is used to predict the 10-year ASCVD risk of the healthy controls in China based on comprehensive indicators, such as age, DM, a history of ASCVD and blood lipid profiles [11]. Our results have showed that most patients with initially diagnosed breast cancer have a low risk of ASCVD. According to different menopausal statuses, there are more moderate-risk and high-risk patients among postmenopausal patients. In this prediction, age is a non-modifiable factor, and the ASCVD risk gradually increases with increasing age. At the same time, the subsequent treatment of cancer patients will further increase the burden on the cardiovascular system. Therefore, as the number of cancer patients increases, it may be more useful to establish predictive cardiovascular risk scores for cancer patients receiving treatment. Therefore, the risk of ASCVD in postmenopausal women should be evaluated when they are initially diagnosed breast cancer.

ALNM is the most common type of metastasis in breast cancer, and it is a major factor in assessing the prognosis. Our study has showed that the place of residence (rural), number of children (≥ 1) and LP (a) level (≥ 500 mg/L) may be risk factors for breast cancer ALNM. A survival study of 18,133 women with malignant tumors in southern China has showed that the prognosis of urban women with malignant tumors is better than that of rural women [28]. Women who had not been pregnant or given birth have a better prognosis than women who have been pregnant or given birth, and some important risk factors for breast cancer are related to fertility [29]. These findings are consistent with our research results. The causes of the poor prognosis in rural women may be related to the fact that they generally have more children, limited access to adequate medical resources and less opportunities to undergo early breast cancer screening. The data supporting the idea that an LP (a) level of ≥ 500 mg/L can increase the risk of ALNM to affect the prognosis of breast cancer patients are limited, and no conclusion can be drawn yet. In the present study, we have showed that patients with breast cancer combined with an LP (a) level of ≥ 500 mg/L may have a higher risk of ALNM. A study has investigated LP (a) levels in patients with breast cancer [30] and it has showed that there is a highly significant correlation between LP (a) level and development and progression of breast cancer. A prospective study [31] has suggested that LP (a) levels are positively correlated with the risks of breast cancer and myocardial infarction. LP (a) may play an important role in the diagnosis and treatment of breast cancer. This is consistent with our findings in this study. The 2016 European Society of Cardiology (ESC)/European Atherosclerosis (EAS) Guidelines for the Management of Dyslipidemia have indicated that people with LP (a) levels of ≥ 500 mg/L have a high risk for ASCVD. And LP (a) level is also strongly correlated with the severity of coronary artery disease. Therefore, attention should be paid to the LP (a) level in patients with breast cancer, which plays a very important role in the prognosis of breast cancer.

## Conclusion

In conclusion, postmenopausal patients have more CVRFs and higher risks of ASCVD than premenopausal patients among patients initially diagnosed with breast cancer. There is a correlation between CVRFs and ALNM in patients with breast cancer.

## Data Availability

The datasets used or analyzed during the current study are available from the corresponding author on reasonable request.

## Abbreviations

FBG: Fasting blood glucose
TC: Total cholesterol
TG: Total triglyceride
HDL-C: High-density lipoprotein cholesterol
LDL-C: Low-density lipoprotein cholesterol
SUA: Serum uric acid
CVRFs: Cardiovascular risk factors
ASCVD: Arteriosclerotic cardiovascular disease
ALNM: Axillary lymph node metastasis
OGTT: Oral glucose tolerance test
SD: Standard deviation
IR: Insulin receptor
ESC: European Society of Cardiology
EAS: European Atherosclerosis
